# Supplementary material for: Blood and tissue biomarker analysis in dogs with osteosarcoma treated with palliative radiation and intra-tumoral autologous natural killer cell transfer
Source: PLoS One. 2020 Feb 21;15(2):e0224775. doi: 10.1371/journal.pone.0224775 (PMC7034869; doi:10.1371/journal.pone.0224775)
Supplement: S1 Table — (DOC) [file pone.0224775.s001.doc]

**S1 Table. List of Canine Specific Primers**

| Primer | Design | Unique Assay ID | Vendor |
| --- | --- | --- | --- |
| CD3E | Intron-spanning | qCfaCID0023499 | Bio-Rad ^a^ |
| CD8A | Exonic | qCfaCED0031325 | Bio-Rad |
| IDO1 | Intron-spanning | qCfaCID0023347 | Bio-Rad |
| IL10 | Intron-spanning | N/A | IDT ^b^ |
| IL6 | Intron-spanning | qCfaCID0020842 | Bio-Rad |
| MKI67 | Intron-spanning | qCfaCID0020352 | Bio-Rad |
| TGFB1 | Intron-spanning | qCfaCID0036443 | Bio-Rad |
| KLRK1 | Intron-spanning | qCfaCID0022821 | Bio-Rad |

^a^ Bio-Rad (Hercules, CA)

^b^ Integrated DNA Technologies (San Jose, CA); synthesized for use in this study using NCBI reference sequence NM_001003077.1 based on published sequences.

Forward sequence: 5’ – ACCACGACCCAGACATCAAGA – 3’

Reverse sequence: 5’ – CCTGGAGCTTACTAAATGCGCT – 3’
