# Supplementary material for: Blood and tissue biomarker analysis in dogs with osteosarcoma treated with palliative radiation and intra-tumoral autologous natural killer cell transfer
Source: PLoS One. 2020 Feb 21;15(2):e0224775. doi: 10.1371/journal.pone.0224775 (PMC7034869; doi:10.1371/journal.pone.0224775)
Supplement: S2 Table — (DOC) [file pone.0224775.s002.doc]

**S2 Table. List of Canine Specific Antibodies**

| Antibody | Fluorochrome ^a^ | Clone | Vendor ^b^ |
| --- | --- | --- | --- |
| CD3 | FITC | CA17.2A12 | Bio-Rad |
| Interferon-γ | AF647 | CC302 | Bio-Rad |
| CD4 | PE/Cy7 | YKIX302.9 | ThermoFisher |
| Granzyme B | PE/APC | GB12 | ThermoFisher |
| CD45 | Pacific Blue | YKIX716.13 | BD Biosciences |
| CD5 | PerCP-eFluor™ 710 | YKIX322.3 | BD Biosciences |
| CD8 | eFluor™ 450 | YCATE55.9 | BD Biosciences |
| 7-AAD (viability) | N/A | N/A | BD Biosciences |

^a^ FITC, fluorescein isothiocyanate; AF, alexa fluor; PE, phycoerythrin; Cy7, cyanine 7; APC, allophycocyanin; PerCP, peridinin-chlorophyll-protein complex; 7-AAD, 7-aminoactinomycin D.

^b^ Bio-Rad (Hercules, CA); ThermoFisher (Waltham, MA) BD Biosciences (San Jose, CA).
